# From Menu to Mouth: Opportunities for Sodium Reduction in Restaurants

**DOI:** 10.5888/pcd11.130237

**Published:** 2014-01-23

**Authors:** Jessica Lee Levings, Janelle Peralez Gunn

**Affiliations:** Author Affiliation: Janelle Peralez Gunn, Centers for Disease Control and Prevention, Atlanta, Georgia.

## Abstract

Restaurant foods can be a substantial source of sodium in the American diet. According to the Institute of Medicine, the significant contribution made by restaurants and food service menu items to Americans’ sodium intake warrants targeted attention. Public health practitioners are uniquely poised to support sodium-reduction efforts in restaurants and help drive demand for lower-sodium products through communication and collaboration with restaurant and food service professionals and through incentives for restaurants. This article discusses the role of the public health practitioner in restaurant sodium reduction and highlights select strategies that have been taken by state and local jurisdictions to support this effort.

## Introduction

Restaurant foods can be a substantial source of sodium in the American diet. In 2010, the Institute of Medicine (IOM) reported, “The current level of sodium in the food supply — added by food manufacturers, foodservice operators, and restaurants — is too high to be ‘safe’ given the chronic disease risks associated with sodium intake for all population segments” (1). Although sodium is an essential nutrient needed in small amounts, it is consumed at levels far greater than what are recommended (2). Excess sodium consumption is a significant health problem that contributes to hypertension, a leading risk factor for heart disease and stroke, both of which are leading causes of death in the United States (1,3). Lower sodium intake can help prevent high blood pressure and control blood pressure when it is already high (4).

## Sodium Exposure in Restaurants

The restaurant industry has a large impact on the provision of food to the US population (1). Data suggest that foods obtained at restaurants contributed nearly one quarter (24.8%) of sodium consumed in the United States during 2007–2008 (5). A 2012 study of 29,531 main entrée menu items served by the top 400 restaurants (by sales) in the United States found that the average sodium content was 1,512 milligrams (6). A 2013 study assessed changes in sodium content in identical fast-food restaurant foods between 2005 and 2011 and found that sodium content increased in 55% of meals assessed (7). The primary sources of sodium intake from restaurant foods are sandwiches, followed by pizza, hamburgers, chicken, Mexican entrées, and salads (1). Furthermore, What We Eat in America 2007–2008 data indicated that the amount of sodium per 1,000 calories is lower for foods obtained from a store (1,519 mg sodium/1,000 calories) than for foods from fast-food or pizza restaurants (1,848 mg sodium/1,000 calories) and sit-down restaurants (2,090 mg sodium/1,000 calories) (5). In 2011, Americans dined out almost 5 times per week on average (8), and annual per capita away-from-home food purchases grew to $2,058 (9).

Although consumers can read and compare Nutrition Facts labels when purchasing packaged foods, acquiring information while eating out can be more challenging. Most restaurant foods and packaged food products sold only to restaurant or food service operations are exempt from mandatory declaration of the Nutrition Facts panel. The IOM recommended in a 2010 report that the Food and Drug Administration require nutrition labeling on food intended for restaurants (1). Restaurateurs can often obtain nutrition information from their suppliers, and public health practitioners can provide assistance on how to ask the supplier and what to ask the supplier for regarding nutrient information on products being considered for purchase. On the consumer side, the passage of section 4205 of the Patient Protection and Affordable Care Act of 2010 mandates calorie labeling on restaurant menus and menu boards for restaurants with 20 or more locations and the provision of additional written nutrition information, including sodium content, on request (10).

Some restaurant operators have already made efforts to provide consumers with more information about the nutrient content of their items. For example, Burgerville, which has 39 locations in Oregon and Washington, began printing calorie, fat, carbohydrate, and fiber content of specific orders on customer receipts as part of a program called Nutricate. In addition, nutrition information is voluntarily made available through in-store kiosks by the chains Au Bon Pain, and Uno Chicago Grill. In 2012, McDonald’s began posting the calorie content of menu items on menu boards in the United States.

In 2011 Darden Restaurants, the world's largest full-service restaurant company, committed to a 10% reduction in sodium over the next 5 years and a 20% reduction over the next 10 years in “items where it has the greatest opportunity to make a difference based on current sodium levels” for US menus (11). In the fast-food sector, between 2008 and 2010, Yum! Brands’ restaurant Taco Bell reduced sodium by 20% in US products, and KFC made substantial reductions in salt in the United States, the United Kingdom, Australia, and New Zealand (12). Pizza Hut, another Yum! Brands restaurant, achieved sodium reductions of up to 50% in core products in Korea, Canada, and Australia but not in the United States (12).

## Opportunities for Sodium Reduction in Restaurants

According to the IOM, the significant contribution to sodium intake made by restaurants and food-service menu items warrants targeted attention to this sector of the food supply. Public health practitioners are uniquely poised to support sodium-reduction efforts in restaurants and help drive demand for lower-sodium products through communication and collaboration with restaurant and food service professionals and through incentives for restaurants. This article discusses the potential role of the public health practitioner in restaurant sodium reduction and highlights select strategies that have been taken by state and local jurisdictions to support this effort.

## Selected Strategies to Encourage Sodium Reduction in Restaurants

### Menu certification programs

In 2012, Wu and Sturm (6) found that restaurants not providing nutrition information at the point of purchase had higher amounts of calories, fat, and sodium on their menus than restaurants that made nutrition information readily available. Many major restaurant chains already require nutrition information from distributors through their contracts and are able to negotiate changes in the nutrition profile of foods they want to purchase. By working with restaurants to encourage promotion of healthful menu items, public health practitioners can help create consumer awareness about sodium in restaurant foods and encourage menu changes to reduce sodium. The Table provides examples of menu certification programs across the United States. The jurisdictions unable to create their own menu certification program could encourage restaurants to increase the proportion of menu items that meet criteria from other programs.

**Table:** Select Menu Certification Programs in the United States

Organization	Program Name	Program Components	Sodium Criteria
Shasta County, California (26)	Healthy Kids Choice	Work with local restaurants to label healthful items meeting the programs nutritional criteria.	≤770 mg per children’s meal
Colorado Department of Public Health (27)	Smart Meal	Work with restaurants to identify options meeting the program’s nutritional requirements through display of a decal on qualifying menu options.	≤1,500 mg per meal and ≤650 mg per side dish
San Antonio Metropolitan Health District (28)	*¡Por Vida!*	Acknowledges menu items meeting select nutritional criteria through use of a menu seal on menus of participating restaurants.	≤750 mg per meal
North Carolina Prevention Partners (29)	Winner's Circle	Provides a logo on participating restaurant menu items for foods meeting its nutritional criteria.	≤1,000 mg per meal and ≤480 mg per side item
The American Heart Association (30)	Heart Check	Allows restaurants to use the Heart Check menu seal on menu items meeting its nutritional criteria.	≤800 mg per meal
Healthy Dining Finder (31)	Sodium Savvy	Labels meals as “Sodium Savvy” if they meet the programs sodium criteria.	≤750 mg per meal; ≤250 mg for appetizers, side dishes, and desserts

### Training and licensure

For restaurateurs to prioritize sodium reduction, they must understand why it is important, where the major sources of sodium are, and that consumers may desire and will purchase lower-sodium options. Food service staff may have varied education and skill levels in sourcing and providing lower-sodium alternatives. Gaps in knowledge can be addressed by developing a training program and requiring participation by new restaurants applying for a license and those renewing their license. A new training module focused on nutrition and sodium reduction could be introduced, or sodium education could be incorporated into existing mandatory trainings, such as those for food safety. Shasta County’s restaurant tool kit, “Cut the Sodium but Keep the Flavor,” provides background information on sodium and why it is a concern and suggests strategies for reducing the sodium content of menu offerings (13).

### Providing subject matter expertise

Often, independent restaurants do not have the same resources, such as a registered dietitian on staff and access to menu analysis programs, as chain restaurants do. Health departments with nutrition support can provide subject matter expertise at no cost to the restaurant. For example, staff in the Schenectady County Department of Public Health Services are partnering with privately owned restaurants in the area to decrease the sodium content in their meals (14). A pilot program found that the partnership provided needed information to restaurateurs about sources of sodium and encouraged sodium reduction in participating restaurants. The Philadelphia Department of Public Health developed and distributed free marketing materials, conducted cooking trainings, and worked with restaurant owners on reducing sodium through the Healthy Chinese Take-out Initiative. They analyzed 2 popular dishes from 20 participating restaurants and found a 20% reduction in sodium. New York City created a brochure for restaurants to assist with compliance when the city’s health code was amended to phase out artificial trans fat in all restaurants and other food service establishments. An online Trans Fat Help Center (15) was also developed by the New York City Department of Health and Mental Hygiene to assist suppliers in their efforts to remove trans fat from their food. A similar Web resource could be created for sodium reduction.

### Incentivizing sodium reduction and providing nutrition information

Local governments have considerable autonomy to enact zoning regulations when there is a reasonable basis for doing so, such as protecting the health of the community. For example, the Mayor of Chicago is considering whether to treat multiple zoning requests from a food retailer as 1 request (ie, only 1 request is needed for the same action at multiple locations) for food retailers opening stores and offering healthful choices in food deserts (16). A similar action could be taken for restaurants that increase the number of healthful choices on their menus.

Modifications to licensure fees, zoning, and other land-use laws may encourage restaurants and food retailers to provide more healthful options. A state or locality could provide incentives, such as reduced licensing fees, for restaurants voluntarily providing more healthful menu options or reducing sodium in their menu items. In this case, it would be necessary to define appropriate and applicable standards or benchmarks. In New York State, Erie County’s “Healthy Choices” menu labeling project offers independent restaurants the ability to create and display Nutrition Facts labels for select menu items at no cost to the restaurant operator through the provision of menu analysis software by the Department of Health and the Western New York Chapter of the State Restaurant Association (17). Maryland’s Healthy Howard also offers free advertising and free nutritional analysis to restaurants certified as healthy (18).

### Creating or joining a Group Purchasing Organization (GPO)

State and local governments typically administer programs and regulate agencies that provide food to the community and may also have a workplace cafeteria. Creating or joining a GPO can increase demand for lower-sodium wholesale items in a given area through increasing the quantity of lower-sodium items that are purchased. GPOs can also decrease costs for members through increasing the volume that is purchased. State and local governments may purchase from the same suppliers as those used by area restaurants, in which case the procurement officers for each can ascertain similar items amenable to purchase by both entities. Entering into a GPO with local restaurants can benefit restaurant patrons and employees and citizens served by the jurisdiction (who are likely also customers of the restaurants). Other organizations in the jurisdiction that may enter into a GPO include hospitals, worksites, and local universities. In the state of New York, some independent restaurants participate in a GPO, the Restaurant Operators Cooperative, affording each restaurant greater buying power than they would have alone while also reducing the cost of ingredients, including those that are lower in sodium. The GPO has allowed participating restaurants to source and procure lower-sodium items that would not have been available or affordable to them otherwise (19).

## Discussion

Efforts can be made in the restaurant environment to reduce sodium exposure and generate greater consumer awareness on how much sodium is in foods consumed away from home. However, certain barriers to sodium reduction do exist. Economos et al reported that a major challenge for independent restaurant owners when conducting nutrition analysis is the lack of standardized recipes (20). Accurate recipe analysis and subsequent menu labeling require a great degree of specificity and can impose cost and time constraints. Concerns about profitability have also been reported as barriers to changing menus (21).

Consumers report that, when dining out, overall nutritional value of the meal is more important to them than individual factors such as sodium (22). However, restaurant customers typically underestimate calorie, sodium, and fat content of restaurant foods (23). Because consumers have less control over the way in which meals are prepared outside the home, it is challenging to simply look at a meal and discern the amount of sodium it contains. Furthermore, options that are popular because they seem healthful, such as salads, can be laden with sodium (ie, ingredients such as cheese, bacon, or croutons are added.) (6). The availability of calorie information on menus or menu boards and other nutrient information upon request may encourage restaurateurs to improve the healthfulness of their menu items, similar to the reformulation of products and reduction in the use of trans fatty acids that was seen after trans fat was added as a component of the Nutrition Facts label.

Given that the menu labeling law affects restaurants with 20 or more locations, the main impact will be on chain restaurants. Food obtained from restaurants is from either an independent or chain restaurant. Independent restaurants are not associated with a national or regional name; therefore, these establishments may have greater flexibility in the types of food served (1). By comparison, chain restaurants are a group of restaurants with the same name and marketing strategy and with standardized menu items across locations; they may be fast food-type restaurants or sit-down restaurants, and they typically serve many more customers and meals than do independent restaurants. Restaurants, regardless of size or type, often use prepared or partially prepared processed foods to develop new menu items. Substantial amounts of sodium may be obtained from processed ingredients used in the development of meals, given that large volumes of food are purchased from distributors. For independent operations, menu decisions are typically made by owners or head chefs and may occur frequently (24). For chains, menu decisions are made at the corporate level and are implemented at each restaurant in the chain (1).

A 2012 study assessed the sodium content of 7 product categories for the 6 largest transnational fast food chains and found sodium content varied across product categories in every country, and individual items marketed as the same product in different countries had very different levels of salt (25). These findings indicate that suppliers are responsive to company needs and reducing salt in restaurant foods is feasible.

Current sodium reduction recommendations focus on gradual reductions of sodium in the food supply to reduce population sodium intake (1). Research suggests consumers may not notice sodium reductions of up to 20%, depending on the food product (1). In addition, as exposure to sodium decreases so can preference for sodium in the diet. However, for gradual sodium reductions to affect population sodium intake without noticeable changes to consumers, reductions will likely need to occur across the food supply and not just in restaurants.

Large restaurants typically have the ability to work with their suppliers to reformulate proprietary ingredients to change or improve the nutritional quality of menu items. Smaller restaurants may not have this ability because of smaller buying power and reach. Independent restaurants may have fewer resources than do chains and may benefit to a greater extent from working with public health practitioners. Consumers’ ability to exercise control over their away-from-home sodium intake is difficult because of the high levels of sodium in restaurant foods. Public health practitioners are uniquely poised to protect the health of the population through efforts such as sodium reduction in restaurants, but appropriate strategies will vary according to the particular needs and characteristics of each community.

The strategies provided in this article are examples of what can be done given the current restaurant food environment and are not an exhaustive list of existing opportunities. Sodium-reduction efforts, especially in restaurants, still encompass a new area of work, and evaluation efforts are ongoing. By supporting sodium-reduction efforts through systems and environmental changes, state and local governments may assist in providing a more healthful food supply, resulting in a healthier population.
